# Augmenting home entertainment with digitally delivered touch

**DOI:** 10.1177/20416695241281474

**Published:** 2024-10-17

**Authors:** Charles Spence, Yang Gao

**Affiliations:** Crossmodal Research Laboratory, 150583 Department of Experimental Psychology, 6396 University of Oxford, Oxford, UK

**Keywords:** digital touch, haptics, multisensory, home entertainment, storytelling

## Abstract

In this narrative review, we take a critical look at the various attempts that have been made to augment home (or personal) entertainment experiences via the addition of some form of digitally controlled tactile stimulation. There has been an explosive growth in the market for home entertainment in recent years, and a majority of smartphones and other wearable electronic devices are now touch-enabled. As such, it is important to consider the challenges and potential opportunities for enhanced multisensory entertainment that may result from the introduction of tactile/haptic stimulation in the context of audiovisual digital storytelling and/or gaming. The key technological, financial (and legal), cognitive, and creative/artistic, challenges associated with the tactile augmentation of home entertainment experiences are outlined. Tactile augmentation, in the sphere of both public and personal entertainment, is more likely to succeed when it goes beyond the merely pleonastic vibrotactile reproduction of those interactions/events than can already be seen and/or heard on screen. At the same time, however, it remains uncertain under what conditions immersion in an entertainment experience will be enhanced by the addition of some form of primitive digital tactile stimulation. Ultimately, until a clear usage case can be made for the benefits of introducing a tactile element to home entertainment, it is unlikely to gain traction and switch from being merely a gimmick to more of a valuable element of multisensory storytelling.

## Introduction

According to a recent industry report, the global home entertainment system market^1^ was estimated to be worth US$ 271 billion in 2023 (Anon, 2023). This figure was predicted to reach US$ 388 billion by 2028 (growing at a Compound Annual Growth Rate of approximately 7.5%). Academic research interest in the field of touch/haptics^2^ has also exploded in the opening decades of the 21st century (see Gallace & Spence, 2014; Parisi, 2018, Figure 1.1, p. 38). However, despite there long having been interest in the possibilities associated with introducing a tactile element into cinema (see Huxley, 1932; and see O’Modhrain & Oakley's 2003 paper entitled “Touch TV: Adding feeling to broadcast media”), it has yet to establish its place as a regular feature of contemporary cinematic experience (see Spence & Gao, 2024, for a narrative historical review). Specifically in the context of touch-enabled home entertainment solutions, legal challenges relating to patent infringements associated with digitally controlled vibrotactile stimulation (in relation to everything from gaming to teledildonics) have hampered the effective development of innovative solutions in this space (see Gallace & Spence, 2014; Parisi, 2018).

In the present narrative review, we take a closer look at the challenges and possibilities associated with various kinds of tactile stimulation specifically in the context of home entertainment. The review is organized around a number of key challenges to the widespread incorporation of tactile (or haptic) stimulation into the home entertainment setting. These challenges are technological, financial, creative/artistic, and cognitive/perceptual in nature. Thereafter, we critically evaluate the research that has attempted to assess the psychological impact of the tactile augmentation of audiovisual digital storytelling. Looking to the future, the increasing ubiquity of touch-enabled mobile and wearable devices in the marketplace would appear to offer a unique opportunity to incorporate a primitive range of vibrotactile stimuli into digital entertainment in the home environment. However, succeeding in this space will likely require a clear analysis and understanding of the fundamental goals of tactile augmentation.

## Commercial Challenges for Touch-Enabled Technologies

The range of touch-enabled technologies developed by human–computer interaction (HCI) researchers for use by people has advanced rapidly in recent years (e.g., Lin & Otaduy, 2008; Obrist et al., 2016a, 2016b; Paterson, 2007). However, very few of the many prototype tactile/haptic stimulation devices that have been developed in HCI labs around the world have actually made it into commercial production. For instance, various innovative prototypes such as tactile gloves, mid-air haptics, and vibrotactile vests have been showcased in research settings but have struggled to reach the market. One notable example is Disney's efforts to develop “Surround Haptics” (Israr et al., 2012; Israr & Poupyrev, 2011; Israr et al. 2011; Surround Haptics by Disney Research, 2015). The system consisted of a basic chair-mounted array of 12 vibratory actuators distributed across a user's back, used as “tactile brushes” to “paint” sensations onto the skin. According to Parisi (2018, p. 345): “The designers claimed that the technology would mean that users do not feel ‘buzzes’ on their body that are common in current haptics technologies, instead they feel smooth tactile strokes moving across their body.” However, there was a lack of convincing empirical evidence to support the claimed benefits in terms of enhancing the gaming experience through “rich and multidimensional tactile feedback.” Consequently, although Disney envisioned that Surround Haptics would have uses in both video-gaming and cinema, the technology was never launched commercially.^3^ In passing, it is perhaps also worth noting that the prototype system does not in any meaningful sense surround the user with haptic stimulation. In fact, it merely stimulates the lower back, backside, and back of the thighs.^4^

A wide range of haptic devices developed in recent decades provide different kinds of tactile stimulation across various skin sites. These can be divided into four broad areas, depending on the site of tactile stimulation. One set of devices provides tactile/haptic stimulation to one (usually the dominant) hand. The mood glove (Mazzoni & Bryan-Kinns, 2016), tactile gloves (Kim et al., 2010, 2015), and mid-air haptics (also known as Ultrahaptics; see Blenkinsopp, 2019; Carter et al., 2013; see also Sodhi, Poupyrev, Glisson & Israr, 2013) all fall into this category. A second class of tactile stimulation solutions is embedded in the user's chair (as in the case of Surround Haptics; Israr et al., 2012; see also Danieau et al., 2012b),^5^ while a third class of solutions involves the user donning a vibrotactile vest or jacket (e.g., Jung et al., 2024; Lemmens et al., 2009; Mazzoni & Bryan-Kinns, 2015; Turchet et al., 2021; see also Dijk et al., 2010; Emerson, 2022). There are also a few examples of thermal and wind/water-based stimulation devices that typically operate at a distance from the user, and which are primarily experienced, or felt, on skin sites that are uncovered (e.g., Cardin et al., 2007; da Silveira et al., 2023; Danieau et al., 2012a; Deligiannidis & Robert, 2006; Han et al., 2018; Hülsmann et al., 2014; Ranasinghe et al., 2018; *SENSIKS*, n.d*.*; Singhal et al., 2023).

**Figure 1.**
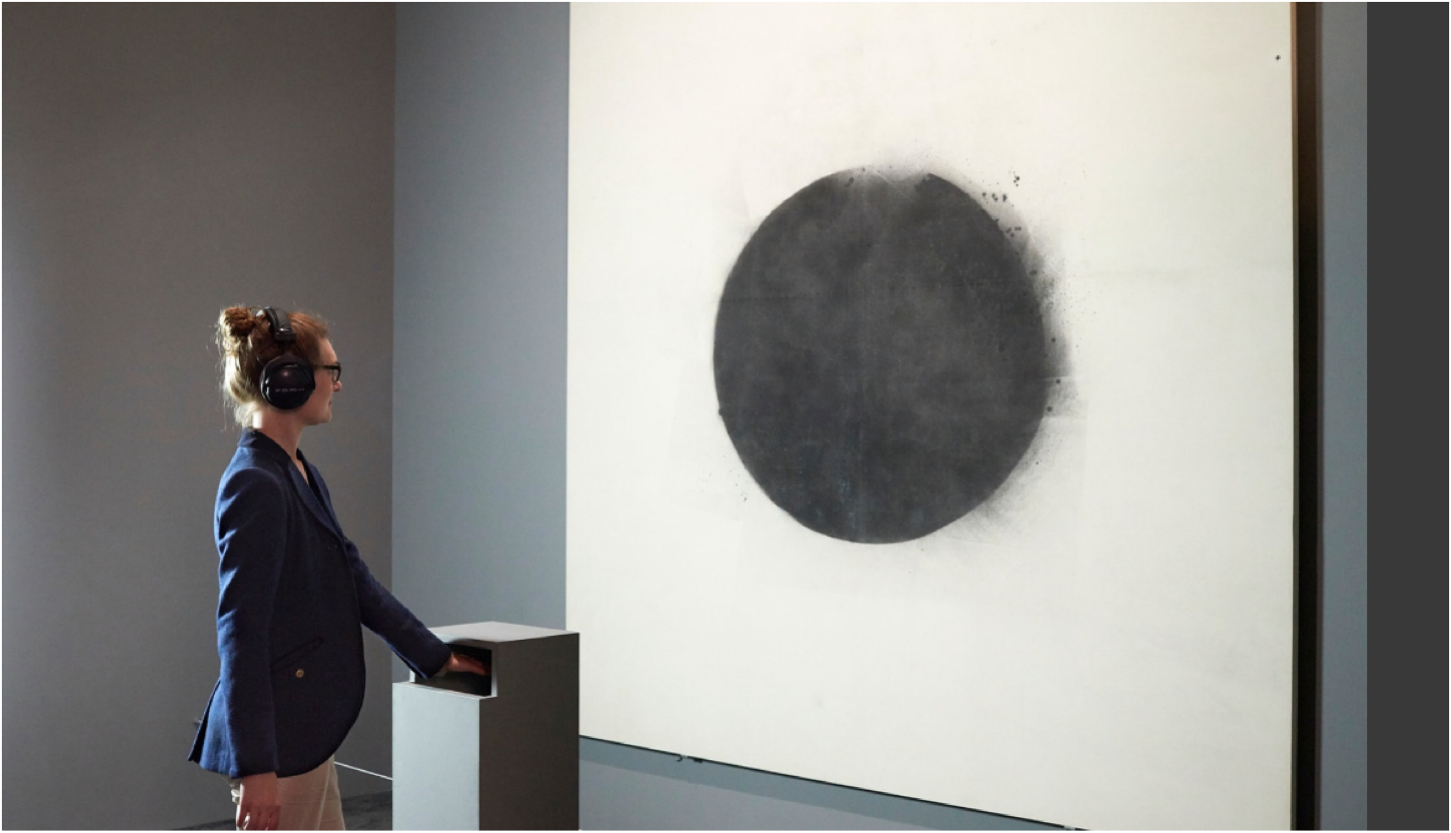
Visitor experiencing mid-air haptic stimulation and sound while viewing John Latham’s (1968) Full Stop at Tate Britain in 2015 as part of the Tate Sensorium exhibition (see Davis, 2015).

Given that the majority of these innovative solutions have yet to make it beyond the HCI research laboratories in which the technologies were first developed, there must remain a question mark over the robustness of these technologies in response to heavy and repeated usage (see Correia et al., 2010; Vi et al., 2017). Specifically, the durability of the materials, issues of delay, and the precision of the mechanisms are all important factors. For instance, one might worry about how long such dynamic devices would stand up to possibly rough and unsupervised use by members of the general public? The one tactile stimulation device that does appear to have achieved some reasonably heavy-duty exposure is mid-air haptics, which uses ultrasound (specifically air pressure waves from an array of ultrasound transducers) to stimulate the skin (typically the hand) without the need for direct contact with the skin (Blenkinsopp, 2019). For example, a mid-air haptic display was introduced as part of one of the exhibits at the Tate Sensorium exhibition in London in 2015 (Flying Object & Chatfield, 2018; Pursey & Lomas, 2018) (see Figure 1). Over a period of six weeks, around 4,000 members of the general public were given the opportunity to experience this mid-air haptic installation while viewing a specially selected work of art from the permanent collection (Vi et al., 2017).^6^ The audience was invited to experience each of four works for 2–3minutes (Pursey & Lomas, 2018).

One challenge when it comes to the commercialization of such haptic technologies is that very different configurations are likely going to be needed depending on the kind of tactile stimulation that one is trying to simulate. For instance, just consider how different the distribution of tactile stimulation over the body surface is that would be needed in order to simulate the feel of a shockwave associated with an explosion (e.g., presumably necessitating the user to wear a vibrating vest or jacket; see Clark, 2018) versus to stimulate (or stand in for) a handshake or the recall when an on-screen character fires a handgun (in which case, a haptic glove, or possibly mid-air haptics might be sufficient). This potential challenge to the use of such tactile technologies assumes that the location at which the tactile stimulation is experienced by the user is not arbitrary. However, it can also be considered that very often the meaning that the user is supposed to associate with the onset of some form of tactile stimulation depends on other simultaneous communication channels rather than the nature of the tactile stimulation itself. For example, users might still understand and feel immersed in an action scene even if the tactile feedback is not perfectly localized (with the action). It might be argued that to the extent that the meaning or reference for a given pattern of tactile stimulation needs to be inferred by the person who experiences the stimulation anyway, the actual location of stimulation might not matter so much. This debate does, though, raise the question of whether tactile/haptic stimulation in such entertainment contexts is experienced from a first person perspective or not—i.e., as when the firing of the gun is felt in the hand that is shown, or inferred to be, holding it.

## Financial Considerations: “How Much Does It Cost?”

Should the technological challenges associated with digital tactile stimulation be resolved then the next challenge concerns the financial aspects of introducing touch-enabled stimulation into the context of home entertainment. At present, it is unclear what proportion of customers would be willing to purchase home entertainment equipment specifically in order to enable them to experience some form of tactile stimulation in the context of digital storytelling or gaming. Indeed, the critical question that few in the HCI community have addressed empirically concerns the public's willingness-to-pay (WTP) for haptically augmented experiences. Réhman et al. (2008) conducted one of the only studies in which the participants were asked whether they would, in fact, be willing to pay for a solution that enabled them to gain some limited information about a live football game through the vibration of a mobile phone motor (in the absence of any other visual or auditory cues). It turned out that the participants were neither willing, nor unwilling, to pay for a solution (though it should be noted that no specific price was placed on the experience). The danger when tactile stimulation is used merely to reproduce pleonastically what is seen on screen or heard (such as when the ball is kicked in a football match that is also visible and audible, say; cf. Réhman et al., 2008)^7^ is that customers will likely not consider the price worth paying (cf. Banes, 2001).

Currently, there is little evidence to suggest that customers are willing to pay for the privilege of tactile/haptic stimulation in the context of home entertainment. One potential solution to decrease costs could be to use existing technologies. The majority of smartphones and other wearable technologies (e.g., Apple watch) already deliver some limited (i.e., primitive) forms of tactile stimulation. This could allow consumers to experience touch-enabled features in home entertainment without incurring additional costs, using the built-in capabilities of their existing devices. However, this raises questions about whether the existing technologies are sufficient or if consumers will prefer to pay a premium for more advanced haptic stimulation solutions.

A related problem relates to patent issues that may impact the cost of producing such tactile technologies for home usage. Indeed, much of the innovation over recent decades has been hampered/stalled due to issues around patent infringement in the area of digital tactile stimulation. According to Murdey (2006), the Immersion Corporation has over 600 patents relating to the technological innovations underpinning touch-enabled devices.^8^ It has frequently been suggested that the many patent infringement lawsuits that the Immersion Corporation has launched may have stifled innovation in the space of haptic entertainment (e.g., Guth, 2003; Osenga, 2014, pp. 460–462). As Fingas (2012) puts it: “Immersion is known for guarding its haptic feedback patents with enthusiasm.” Similar challenges have also stymied developments in the field of “teledildonics” (Mullin, 2015; see also Clark, 2018), which constitutes another form of home entertainment (Strong & Gaver, 1996). In those cases where these patent issues have been successfully (if not necessarily amicably) resolved, the licensing of the underpinning haptic technologies by the companies concerned is likely to elevate the price of touch-enabled solutions for the home environment. Consequently, this is likely to raise the financial bar for the experiential value that such devices need to deliver in order to become commercially viable.

## Creative Challenges: “Why?,” and “How?”

Should the relevant technology be produced that is suitably robust for regular home usage and also available at a price that customers are willing to pay for, then the next challenge becomes one of standardization. In particular, the need for industry-standard operating protocols to deliver tactile stimulation (Parisi, 2018) opens up creative possibilities. There needs to be some sort of haptic API/codec that designers/creators could access to facilitate the production of touch-enabled content (cf. Israr et al., 2012), leading to creative storytelling and immersive experience. This would presumably also necessitate some degree of conformity with regards to where the tactile stimulators are likely to be positioned on the user's body. Given the challenges that are normally associated with standardizing technology (consider, e.g., the battle between VHS vs. Betamax in the late 1970s and 1980s), this is again where the ubiquitous availability of touch-enabled smartphones and other mobile devices may come to the fore. Illustrating the potential problem of haptic augmentation specifically in the context of gaming, Parisi (2018, p. 345) notes how: “Almost without comment, Microsoft added two additional motors to its Xbox. One controller (‘impulse triggers’), but game developers have been slow to code vibrations specifically for the extra motors, leaving the new resource untapped.”

Consider, for example, Apple's “taptic engine,” which, at least according to commentators, can mimic the feel of buttons, taps, and simulated heartbeats. Crucially, Apple's Taptic API allows developers to create apps with their own haptic features (see Apple, n.d.; Clark, 2018).^9^ Given such developments with regard to the development of standardized protocols, it becomes possible to start thinking about the development of what some researchers have termed a “feel effect” library (Israr et al., 2014; Zhao et al., 2014), which will enable designers and directors to deliver a range of haptic effects to augment audiovisual content. The hope is that such developments might one day enable designers/directors to deliver a small range of (relatively primitive) yet differentiated haptic/tactile effects to augment the audiovisual content they deliver (see also Waltl et al., 2012). Here, one might also consider the automated extraction of key features or events in music videos (such as loudness, pitch, etc.), or when live-streaming sporting content, that could potentially be augmented by tactile stimulation (cf. Best, 2022; Réhman et al., 2008).

Assuming that the means now exist to achieve the goal of delivering a meaningful range of tactile stimuli, the creative challenge lies in determining what effect the designer/creator would like to elicit/evoke by stimulating the user's skin. In particular, by using such a library to deliver simple vibratory stimuli to the hand that is likely holding the device on which the content is being viewed. An important secondary question here is why touch should be considered as the best or most appropriate channel through which to deliver such effects. Indeed, unless the impression that touch has been added merely as a “gimmick” can be shaken off, it is unlikely that it will become a viable commercial proposition. Furthermore, and as has been mentioned already, it would seem unlikely that the merely pleonastic use of touch in an entertainment context will ever succeed (cf. Banes, 2001; Spence, 2020).

### Why Introduce Haptics to Home Entertainment?

Banes (2001) highlighted the various ways in which olfactory cues can be used effectively in the context of live performance including the evaluative, the critical, and the humorous (cf. Spence, 2021). Banes also notes how scent (like sound) has sometimes been used/introduced for its contrastive function with the action that is taking place on screen/stage, for example, playing slow music when a chaotic fight breaks out (see also Boswell, 2012; McGinley & McGinley, 2018).^10^ Scent can evoke memories and trigger nostalgia. It is, however, not immediately apparent how this might play out in the context of digitally delivered tactile stimulation (though see “Hands-touching-hands” in Gallace & Spence, 2014).^11^ Indeed, we are not aware of anyone having created a tactile joke yet! Adding scent to live performance can also sometimes help to break the so-called “fourth wall” (McGinley & McGinley, 2018; Wilson, 2020). Finally, Banes highlights various instances where the introduction of scent in the live performance setting has more of a ritualistic connotation. It makes sense to consider whether any of the same outcomes can be achieved by stimulating the skin in the context of home entertainment. However, the immediate challenge here is that there is normally little semantic meaning attached to passively delivered tactile stimulation; this obviously contrasts with the case of scents that have often been reported to trigger autobiographical memories, as well as a range of other associations (see Spence, 2020, for a number of such examples).

At this point, it is interesting to look at how digital vibrotactile stimuli are used elsewhere, in order to know what the possibilities and limitations might be as far as home entertainment is concerned (e.g., see Park et al., 2013).^12^ One of the primary uses for digitally controlled vibrotactile stimulation currently is in the context of warning/alerting (e.g., for car drivers; see Ho & Spence, 2008; Meng & Spence, 2015). In this case, the simple sudden onset of vibration automatically alerts the driver that something needs their attention. Another application is in video games, such as in Nintendo's *Luigi's Mansion 3* (Next Level Games, 2019), where different vibration intensities provide hints to players about their proximity to ghosts, thereby enhancing the gaming experience by integrating sensory feedback directly relevant to game objectives (see Gao & Spence, 2024). Meanwhile, in the case of mobile devices, the vibration typically indicates an incoming message (without the need for an auditory alert). The research shows that people typically find it hard to discriminate between different kinds of passively delivered vibrotactile stimulation (see O’Modhrain & Oakley, 2003, on the distinction between active and passive haptic stimulation in the setting of home entertainment; cf. Lederman & Klatzky, 1993; Myles & Binseel, 2007). It is interesting to note how tactile/haptic feedback sometimes appears to elicit qualitatively different responses than if the same information were to have been presented via a different modality (see Manshad & Brannon, 2021, for an intriguing example).

Relevant here, consider Réhman et al.'s (2008) attempt to convey where on the football pitch the ball was, and sometimes even which team was in possession too, when people were holding a mobile device. Given the fact that all vibration was presented from a single offset motor, these five categories of information were linked to one of five discriminably different rates of vibration (of the offset motor in a mobile phone). Obviously, such an arbitrary mapping is not going to be particularly intuitive, and hence requires training in order to minimize errors when users try to interpret the temporal haptic patterns, which introduces a learning curve for the users. This will likely also increase the cognitive demands on the user (see Spence & Driver, 1997), and may act as a barrier to uptake, especially if the literature on sensory substitution is anything to go by (see Spence, 2014, for a review). Israr et al. (2014) have also made some initial steps towards the development of a small number of semi-intuitive tactile stimuli for use with a haptic vest. These included the semantic categories of “rain,” “travel,” “strike,” “brush,” “pulse,” and “motor sound” that were associated with different patterns of tactile stimulation varying in terms, of their duration, intensity, and stimulus onset asynchrony between stimulation.^13^

### Diegetic and Non-Diegetic Use of Haptics

A decade ago, Danieau et al. (2014) put forward a taxonomy of haptic effects. They drew a parallel between the possible uses for haptic effects in cinema and the way in which audio is used in the context of movies. For instance, sound is sometimes used not only to enhance realism (e.g., as in the case of sound effects) but also to create ambiance (as in the case of mood music; see Frith, 1984) in film. These two categories of audio content are known as diegetic and non-diegetic sound, respectively. The former refers to a sound for which the source belongs to the diegesis (that is, the recounted story). By contrast, in the case of non-diegetic sounds, the sound source is neither visible nor implied by the on-screen action. Examples of the latter include such things as a narrator's comment or mood music (MasterClass, 2021).^14^ One other class of sound is trans-diegetic: This is where the sound starts as part of the story and then switches to become part of the background, or vice versa (MasterClass, 2021).

Danieau et al. (2014) suggested that haptic effects in the context of touch-augmented cinema can also be classified as either diegetic or non-diegetic. The former may help to enhance physical events in the audiovisual content in a similar manner to how haptic effects have been used in virtual reality (VR) applications (see Gallace et al., 2012). According to Danieau et al., diegetic haptic effects can either be local or global: Local effects are associated with a specific object in the scene: for example, force-feedback (O’Modhrain & Oakley, 2003) or vibrations related to events occurring with an onscreen character or vibrations representing the position of the ball in a soccer game (cf. Réhman et al., 2008). Global effects relate to the environment, such as the vibrations associated with an earthquake in a movie^15^, or a system that allows users to touch the objects within a scene (Cha et al., 2009). Thermal, wind, and water effects might also fall into the latter category. Akin to the auditory example, non-diegetic haptic effects refer to those elements that are not attached to the fictional world depicted by the story.^16^

**Figure 2.**
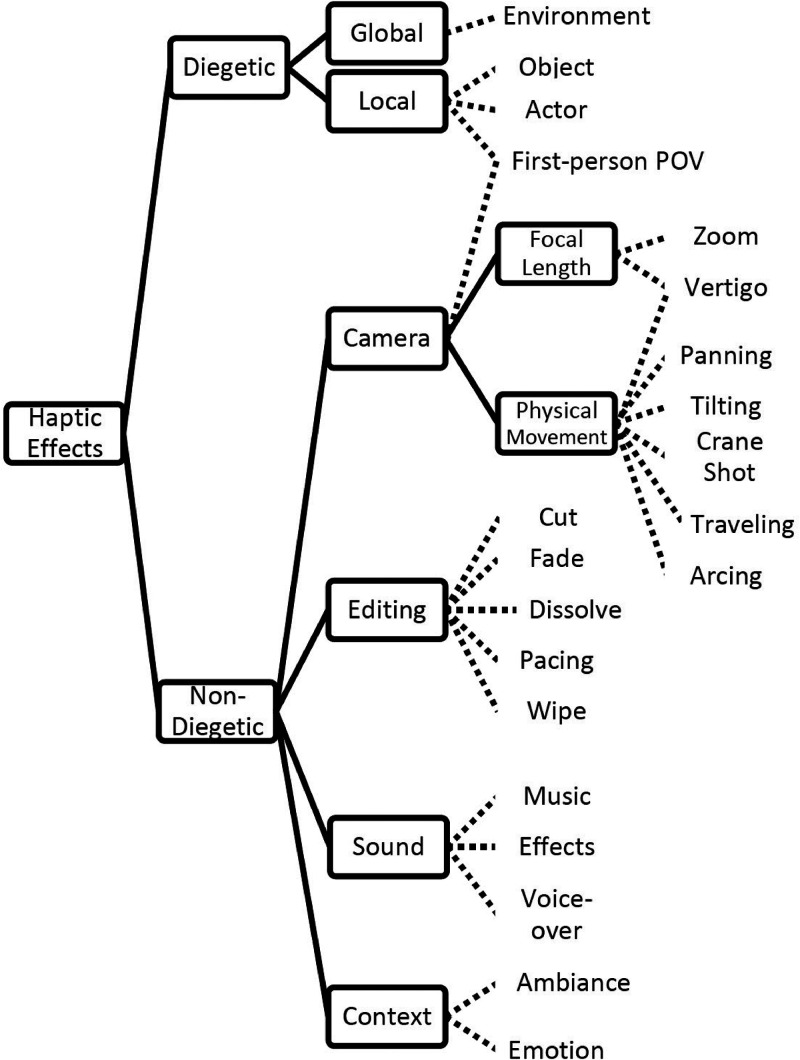
Diegetic and non-diegetic uses of haptics in the context of augmented cinema as suggested by Danieau et al. (2014).

Danieau et al. (2014) suggested linking specific cinematographic techniques with particular patterns of vibration delivered via an augmented chair (see Figure 2 for their suggested breakdown of haptic effects in film/cinema). Going one stage further, we are certainly not aware of any examples of the tactile/haptic augmentation of entertainment that fit into the trans-diegetic category. It is an interesting to consider whether the mid-air haptics introduced as part of the Tate Sensorium exhibit in 2015 (see Figure 1) should be classified as diegetic or not? According to Pursey and Lomas (2018, p. 358), the ultrahaptics device in this installation was programmed to create: “a sensation of placing one's hand into a large area, or over a horizon, before peppering the hand with individual dots, a reference to the spray paint. Sound (via headphones) was synchronized with this and provided a deep, epic whistling sound, before again reflecting the idea of small units hitting the canvas.”

One final point to consider here concerns those situations in which the tactile augmentation of entertainment happens to have been added by someone other than the creator of the content itself. Should the tactile/haptic augmentation successfully influence, or change, the meaning of the experience for the user/viewer (cf. Pozo, 2016, for one example in the context of touch-augmented film experience) then one might want to question whether it is ethical to modify the director's/creative's intentions (i.e., by adding tactile stimulation post-production). While this is admittedly less likely to be an issue in the context of gaming or popular culture, this very issue was raised as part of academic discussions following the very successful Tate Sensorium exhibition in 2015 (see Pursey & Lomas, 2018; Vi et al., 2017).

It is certainly possible to express and decode emotion through touch and vibration (Réhman, 2010). However, the emotion that is delivered by digital technology is typically always indirect (i.e., inferred). As such, the immediate and direct emotional benefits that are conveyed by the slow warm stroking of the C-tactile afferents in the hairy skin that have been documented are unlikely to be observed (see Gallace & Girondini, 2022; Hertenstein et al., 2009; Hertenstein et al., 2006; Spence, 2022). Nevertheless, there is a HCI literature on which to draw around affective haptics (Eid & Osman, 2016; Gatti et al., 2013; Huisman & Frederiks, 2013; Tsetserukou et al., 2009). One further issue that has yet to receive much attention from researchers concerns the possible confusion that people may experience about what any particular tactile/haptic stimulus is supposed to indicate/represent. Presumably nothing in the parameters of the sensory stimulation itself (other than perhaps its synchronization with the audiovisual stimuli; though see Ablart et al., 2017a) will indicate whether a particular tactile stimulus is diegetic or not?

## Cognitive, Attentional, and Perceptual Considerations: “What Will People Feel?”

**Figure 3.**
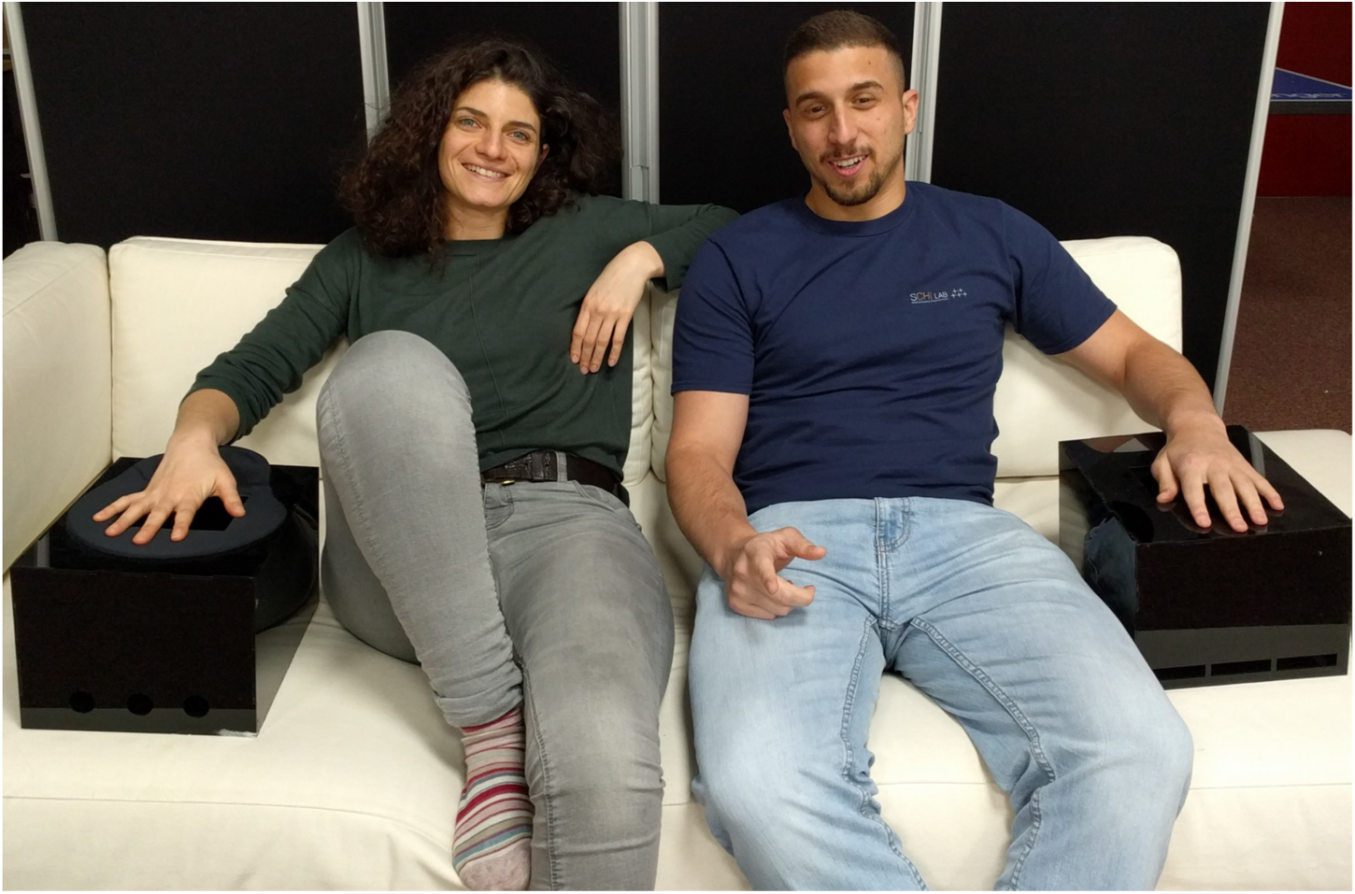
Does perspective/viewpoint matter? According to this image, as presented in Vi et al. (2017), the same mid-air haptics stimulation is provided to the right (or left) hand but this may lead to conflict depending on the nature of any other sensory stimulation (e.g., what is seen on screen) that is delivered.

Should the technological, financial, and creative issues associated with the tactile augmentation of home entertainment be resolved, then the final challenge to consider here concerns the information (or cognitive) processing bandwidth of the user (see Gallace et al., 2012). There are also potential issues related to the ubiquitous phenomenon of sensory (typically visual) dominance (Gallace & Spence, 2014; Hutmacher, 2019; Spence et al., 2001). While it is often claimed that adding a tactile dimension to VR applications can help to enhance the user's sense of presence and immersion in the action (e.g., Hoffman, 1998; Sallnas et al., 2000), the possibility of attentional distraction also needs to be carefully considered (see Gallace & Spence, 2014; Vi et al., 2017). Indeed, this is likely to be an especially pertinent issue when the meaning of any tactile stimulation needs to be discriminated and/or decoded (which is mostly always the case). Bear in mind here also the issue of viewpoint, or perspective, when people passively view content (cf. Zhang et al., 2022). Tactile augmentation that is targeted at a hand is typically designed for the dominant hand. However, that leaves open the question of whether left-handers would struggle (see Figure 3). An important distinction worth considering here is how VR-gaming is normally presented from a first-person perspective (and the viewer is the main character) and haptic feedback is active (note here that haptic perception is inherently active, with touch being a process that is closely coupled with, even mediated by, physically interactions or events). By contrast, different perspectives are used in cinema (Danieau et al., 2014), and the viewer does not direct, or control, the action, meaning that any tactile/haptic stimulation is more likely to be passive (see also O’Modhrain & Oakley's, 2004, investigation of haptic cues in the context of children's cartoons), except in cases of interactive films like *Black Mirror: Bandersnatch* (Slade, 2018), where the viewer actively makes choices that influence the story.

### Navigating the “Uncanny Valley” of Haptics

One of the perceptual challenges associated with tactile augmentation in a digital entertainment/education setting concerns the so-called “uncanny valley of haptics.” The surprising observation that has emerged in recent years is that enhancing the realism of digital tactile stimulation can sometimes result in a feeling of unease amongst users, as the pattern of tactile stimulation becomes increasingly, but never quite fully, realistic (see Berger et al., 2018; Desnoyers-Stewart et al., 2024). Similarly, the use of more realistic touch in VR training application has been reported to “ruin” the sense of immersion (Barad, 2018). Given such observations, some commentators have argued that it may make more sense to deliver just enough digital haptic stimulation to ensure a wider uptake of haptic content in the years ahead (Barad, 2018; Pacchierotti & Prattichizzo, 2023). According to such a view, developers should focus their efforts on designing simple tactile/haptic stimulation opportunities that are capable of providing the kinds of feedback, or information, that are truly important for the task, or content, at hand. At any rate, the existence of the uncanny valley of haptics means that the hyper-realistic feel of the bearskin rug that was anticipated in Huxley's 1930s futuristic vision of “the feelies,”^17^ as mentioned in his novel *Brave New World,* is unlikely ever to be realized.

Avoiding the uncanny valley of haptics may, however, not be something that one actually needs to worry about if the offset motors in smart phones etc. are what end up being used to deliver tactile stimuli to the user (given the primitive range of vibrotactile stimuli that such technology can deliver; Kaaresoja & Linjama, 2005). At the same time, it is perhaps also worth bearing in mind how consumers traditionally appear to have been far more willing to pay for enhanced visual resolution for their digital devices than to splash the cash on better sound quality (consider the case of laptop computers, where screen resolution always seems to win out over sound quality). The same asymmetry is presumably also likely to occur when it comes to enhancing the quality of tactile stimulation in the context of digital home entertainment. Once again, therefore, what this means in practice is that the problem of the uncanny valley of haptics may not be a relevant issue in the context of touch-enabled digital home entertainment anytime soon.

### Information Processing Bandwidth and Sensory Dominance Considerations

Originally writing in 1955, Morton Heilig (1992), one of the inventors of multisensory VR, suggested that the sense of touch has far less ability to capture the attention of the cinema audience. A range of other neural/information-processing metrics similarly place touch behind vision (see Table 1). According to Kaczmarek et al. (1991), if presented properly humans can process information through the sense of touch at the rate of 2–56 bits/second (see also Coulton, 2017). The fact that the bandwidth of the skin is so much lower than for vision presents challenges to the effective communication of information through the skin (see Gallace et al., 2012).

**Table 1.** Summary of the number of sensors, number of afferents, information transmission rates/channel capacity (from Zimmerman, 1989; see also Nørretranders, 1998), % of attentional capture (from Heilig, 1992), and % of neocortex (Felleman & Van Essen, 1991) relative to each sensory modality.

Sensory system	Number of sensors	Number of afferents	Channel capacity (bits/s)	Psychophysical channel capacity (bits/s)	% Attentional capture	% Neocortex
Vision	2 × 10^8^	2 × 10^6^	10^7^	40	70	55
Audition	3 × 10^4^	2 × 10^4^	10^5^	30	20	3.4
Touch	10^7^	10^6^	10^6^	5 (−8)	4	11.5
Taste	3 × 10^7^	10^3^	10^3^	1 (?)	1	0.5
Smell	7 × 10^7^	10^7^	10^5^	1 (?)	5	N.A.

*Note*. Adapted from Gallace et al. (2012).

It is interesting to note how some of the most impressive uses of tactile/bodily sensation occur under those conditions where the dominant visual sense is temporarily removed (cf. Delaunay, 2014). This may help to address issues related to visual dominance (Danieau et al., 2014). Along conceptually similar lines, Tao et al. (2021) studied the effects of vibration on assisting gameplay and improving player engagement under those conditions where the sound was absent. Researchers have also been interested in the use of touch (a vibrating jacket or harness) to enhance the music experience of deaf (as well as regular hearing) individuals (Best, 2022; Gunther & O’Modhrain, 2003; Hayes, 2015; Turchet et al., 2021). At the same time, however, it is also important to note how the less intuitive the tactile stimulation happens to be (Réhman et al., 2008), and hence the more it needs to be interpreted by the user, the higher their cognitive load is likely to be (i.e., for whoever is trying to enjoy the content).^18^ Indeed, given that the audience's tactile experience is never going to precisely match what is being depicted on screen, there is a danger that bodily stimulation might simply end up removing people from the action rather than immersing them in it (as intended).^19^ Relevant here, prior research on vibrotactile warning signals for car drivers has highlighted how the passive delivery of tactile stimulation tended to draw the driver's attention towards their own body, rather than to the peripersonal region of space where the relevant danger happened to be located (Ho et al., 2006). In the context of film, one might similarly worry that stimulating the viewer's body serves to take their attention away from the screen.

Another important, if understudied, issue concerns the problem of perspective alignment when it comes to tactile stimulation (Zhang et al., 2022). While auditory and olfactory stimulation might generally be assumed to be accessible to/experienced by anyone who is depicted in the scene, touch is different. While a viewer might expect any global haptic effects (such as those associated with rain, heat, or an earthquake to be associated with everyone depicted in a scene, local haptic effects (such as the feel of an orange that a character has picked up) would only be experienced by the character holding the fruit, and not by anyone else who may be present in the scene. As such, delivering local tactile/haptic feedback requires a commitment to adopting the viewpoint of a particular character in a scene (cf. Spence & Gao, 2024), unless it is used in an interactive film where the viewer can choose to be the protagonist from the start.

### Synchronization Issues

Synchronization issues have long bedeviled the successful roll-out of multimedia technologies (e.g., ITU-R BT 1359-1, 1998). While historically such issues have primarily been considered in the context of audiovisual synchronization, they are presumably equally likely to adversely affect touch-enabled multisensory entertainment solutions in the home as well. Synchronization may be particularly challenging in the context of the haptic augmentation of live-streaming of sporting events, say (Réhman et al., 2008). That said, it is currently unclear how precise the synchronization of tactile stimulation needs to be (Vi et al., 2017), or how wide the effective temporal binding window is, when streaming multimedia content on such devices in the home environment (see Spence et al., 2001, for guidelines based on highly controlled laboratory research with simple stimuli in the absence of any crowding, or distractors).

As soon as any haptic stimuli are synchronized with audiovisual content (Danieau et al., 2013), then it suddenly becomes important to recognize that what the user sees and/or hears may also change what they feel (e.g., see Lee et al., 2009; Lee & Spence, 2008a, 2008b, 2008c). For instance, some years ago we demonstrated how the two-flash illusion (Shams et al., 2000) could be extended to enhance the dynamic range of perceived tactile stimulation as delivered by the activation of a touchscreen. In particular, the touchscreen was perceived by the user as a single tactile stimulus when a unitary auditory or visual event was presented simultaneously. However, it was mostly perceived as a double vibration if two (or more) visual or auditory stimuli were presented at the same time. What this means, in practice, is that those wanting to augment the tactile channel may need to pay careful attention to whatever stimuli happen to be presented in the other senses.

## Experimental Assessment: “What Impact Does It Have?”

Is it really possible to enhance storytelling with haptic feedback (Clark, 2018; Israr et al., 2014)? Is immersion and/or presence really the most appropriate measure of success in the context of touch-augmented home entertainment, or would Quality of Experience (QoE) measures (Jain, 2004) provide a more appropriate means of assessing the impact of adding touch/haptics? Given the various challenges that have been identified so far in this review, one can ask whether any successful usage cases have been developed whereby the addition of haptic effects has clearly enhanced the user experience in an audiovisual digital entertainment (e.g., storytelling or gaming) context. An additional question here is to identify the kinds of benefit (e.g., to the user's sense of presence, their immersion, their emotion, and/or their interpretation of the narrative).^20^

Lemmens et al. (2009) developed a number of tactile patterns for a haptic jacket based on typical touch behaviours from human emotional touch communication (e.g., highly energetic movements to indicate surprise or happiness) as well as based on common wisdom and sayings (e.g., “butterflies in your stomach”). Those patterns were presented together with short movies. Users’ reactions (*N* = 14) were assessed through physiological measurements (respiration, heart rate, skin conductance level) and questionnaires (self-assessment manikin and immersion questionnaire). The results suggested a positive effect of haptic stimuli on peoples’ immersion in the 7 film clips selected to be associated with a different emotion (namely, love, enjoyment, fear, sadness, anger, anxiety, and happiness), with the respective clips being extracted from the movies Braveheart, When Harry met Sally, Jurassic Park III, The Lion King, My Bodyguard, Silence of the Lambs, and a Tom & Jerry cartoon. However, only a single haptic condition was used per movie, thus making any comparison between the designed haptics and other approaches impossible.

Danieau et al. (2014) conducted a study in which they compared the effects of cinematic tactile stimulation (mirroring the movement of the camera) delivered via a vibrating chair (see Figure 2) to semantic tactile stimulation (designed to mimic strangeness as in the Dutch Angle when the viewer sways from side to side, arcing, tilting, and vertigo associated with particular camera movements), random tactile stimulation and a baseline no stimulation block. The 38 participants who took part in this study viewed a large number of 7-second clips. The results revealed that the cinematic touch condition led to a significantly higher QoE rating (almost 0.3 higher on a scale that had been normalized between 0 and 1) than when no stimulation was presented (see Jain, 2004). The lowest QoE ratings were associated with the random stimulation condition (see Table 2).

**Table 2.** Summary of the results of those studies that have attempted to assess the impact of adding a tactile/haptic element to film. Note the short length of clips used, and also the wide variety in terms of the tactile/haptic stimulation devices used.

Study	AV stimuli	Tactile	*N*	Assessment measure	Result
Lemmens et al. (2009)	2 × 7 Short movies	Haptic jacket	14	Self-Assessment Manikin Immersion questionnaire and 4 physiological measures	Adding tactile stimuli had a positive effect on immersion
Danieau et al. (2014)	7-second film clips	Hapchair-vibrating chair	38	Quality of experience (QoE)	Adding cinematic tactile effects significantly increases QoE
Mazzoni & Bryan-Kinns (2016)	2 × 12 29-117 second film clips	Vibrating mood’ glove	21	Valence and arousal	Adding tactile stimuli significantly inceases arousal
Ablart et al. (2017a, 2017b)	6 × 1-minute movies	Mid-air haptics	2 × 13	Valence and arousal	Adding tactile stimulation slightly, but significantly increased arousal
Vi et al. (2017)	John Latham painting plus soundscape	3 Mid-air haptic patterns	>2000	Questionnaire and skin conductance (not analyzed)	Adding 2 of haptic patterns led to significantly higher arousal than to the 3rd pattern (but not compared to no haptics or baseline)
Ranasinghe et al. (2018)	4 × 30-second VR film clips	Wind & temperature	20	Sense of presence, heart rate, and skin conductance	Adding tactile stimulation to VR significantly increases sense of presence

Mazzoni and Bryan-Kinns (2016) sent subtle tremors through a mood glove in a number of short movie clips. This heightened the viewer's emotions, accentuating the suspense, exuberance or terror that was playing out on the screen. Mazzoni and Bryan-Kinns selected a dozen scenes from three movies (the surreal fantasy Edward Scissorhands, the French comedy Amelie, and the psychological thriller Memento) to capture a range of potential moods. A group of 21 participants each watched a total of 24 short clips (lasting for between 29 seconds and one minute and 57 seconds) with and without the vibrating glove and also with the soundtrack present and absent, rating their emotional response in each case. Eight vibrators were mounted in the palm and back of the glove. Although the haptic stimulation presented from the glove didn’t affect the participants’ feelings of positivity or negativity (i.e., valence) it did increase their sense of arousal (resulting in a more intense mood perception of the associated film scene). Low intensity and low frequency vibrotactile stimulation was associated with calmness, whereas low intensity but high frequency vibrotactile stimulation heightened tension. According to Dr. Nick Bryan-Kinns: “The vibrations are not mimicking what's on the screen, they’re enhancing emotions and that's the unique element here. It's like writing a musical score” (quoted in Daily Mail Reporter, 2016). The suggestion here, then, is that the tactile/haptic stimulation was more than merely pleonastic (cf. Banes, 2001), and was perhaps playing a non-diegetic role equivalent to that achieved by the incorporation of mood music in film.

Ablart et al. (2017a, 2017b) conducted a study in which two groups of 13 participants watched six one-minute movies that could be augmented with tactile feedback (mid-air haptics) or not, and that was either presented in synchrony with the action or not (synchronization varied between-groups). Analysis of the skin conductance responses (SCRs) of a group of participants to the film clips provided cues as to the most arousing points of the movies (linked with peaks in SCR, that were used to present the haptic patterns). Thereafter, the mid-air haptic pattern consisted of a single point displayed on the hand that changed location every 100 ms, following a pseud-random pattern on a 5 × 5 cm grid. The pattern was presented for the 3 to 5 highest SCR peaks in the clip. The results showed that any kind of tactile stimulation increased participants’ self-reported arousal levels (activity) but had no significant effect on valence (positive/negative). Surprisingly, however, desynchronizing the tactile stimulation did not alter the results. Given the efforts required to determine when to present the tactile stimuli, and the fact that it required a special device to deliver the ultrahaptics stimulation, this would not appear to provide good grounds for adding touch to movies, contrary to some of the press headlines associated with the study.

Vi et al. (2017) conducted a study involving a three-minute exposure to John Latham's (1968) painting Full Stop as part of Tate Sensorium exhibit. In total, more than 2,000 people were quizzed about their experience. Interestingly, the visitors were exposed to one of three different haptic patterns (an expanding circle *N* = 1889, two dots rotating in a circle *N* = 133, or a linear translation of a dot across the hand *N* = 152) using a mid-air haptic display. While the results were generally positive,^21^ adding either of the first two haptic patterns led to higher arousal (as assessed by responses to the question: “How intense was the multi-sensory experience created for this painting?”) than the linear pattern (0.3 on a 5 point response questionnaire). However, visual liking was equivalent as was people's liking of the multisensory experience across the three haptic conditions. Unfortunately, there was no baseline painting only or painting plus sound condition to determine what effect, if any, was specifically linked to the haptic stimulation.

**Figure 4.**
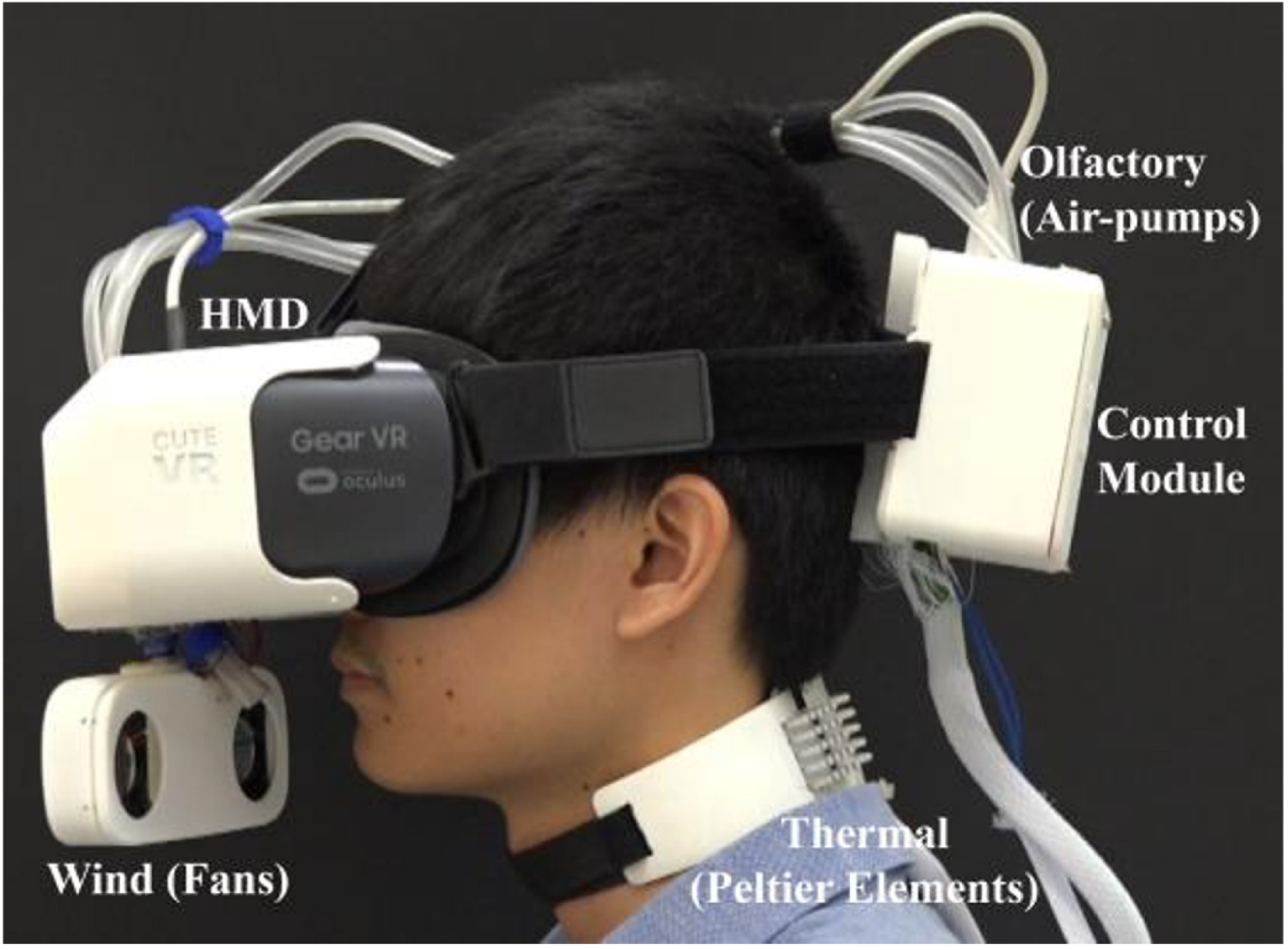
Other devices for delivering wind and thermal (and olfactory) effects would seem wholly impractical, not to mention uncomfortable for prolonged (and unsupervised) use (Ranasinghe et al., 2018).

Ranasinghe et al. (2018) studied Season Traveller, a multisensory VR narration of a hot air balloon journey through four seasons within a mystical realm (spending about 30 seconds in each). Four short scenarios that depicted the four seasons were modelled. Temperature, wind, and olfactory cues were presented throughout each scenario. A total of 20 participants experienced the four seasons in five different sensory configurations (involving audiovisual plus some combination of haptic—thermal and wind—and olfactory cues) giving their response in terms of subjective measures of presence. The participants’ heart rate and electrodermal activity were also measured. Crucially, adding the wind and thermal effects to the audiovisual plus olfactory experience led to a significantly higher rating (of immersion) from participants. That said, the device itself looks like it may have been rather uncomfortable to wear, especially for longer periods of time (see Figure 4).

Clark (2018) reviewed several intriguing storytelling experiences that had engaged the tactile/bodily senses. In one such experience, called HERO, an immersive war-zone themed multisensory experience, with an element of role-play used a VR headset together with a haptic backpack that transmitted vibrations synched to the explosions in the simulation, as well as some real tactile props and ambient sounds. This prize-winning immersive multisensory experience, described as offering an “astonishingly immersive experience,” reflects a combination of both analogue and digital tactile stimulation (Starbreeze, 2018).^22^ Meanwhile, another touch-augmented experience, Space Explorers, involved a person sitting on a chair that could spin 360°, as well as pitch both forward and backward by 35 degrees (it is, though, hard to see that people at home would necessarily be convinced to pay for such an experience). The chair also incorporated elements of haptic feedback and spatial audio to complement the VR headset and enable the viewer to experience various journeys by different modes of transport (plane, snow rover, etc.).

One other multisensory VR experience is *In Pursuit of Repetitive Beats* (2022) by Director Darren Emerson, which was designed to recreate the club scene of Britain in the 1980s. This experience won multiple awards and has been featured at various film festivals. The experience lasts about 35 minutes and involves the use of VR headsets, haptic vests and controllers. The haptic vests enable users to feel the vibration that stimulates the intense bass of rave music and wind effects mimic the swift movement through different rave scenes, enhancing the overall sense of immersion (Baldwin Pask 2023; Nguyen, 2023). Audience feedback highlights the powerful emotional impact of the experience, with one audience moved to tears, and another emphasizing how VR and sensory technology create a very engaging environment (East City Films, n.d.).

One particularly intriguing example of the use of mid-air haptics comes from the independent VR studio Fallen Planet (Blenkinsopp, 2019). They created an immersive, three-minute multisensory haptic horror experience called *AFFECTED: The Visit*, that integrated mid-air haptics*.* An especially impressive aspect of the experience is that users got to experience an Ouija board. This is a particularly clever use of haptic feedback because the otherworldly feel is one of the few experiences that it is simply not possible to show visually or auditorily. Hence, this use of local tactile feedback avoids the criticism that has been levelled at certain other touch-enabled content that it is merely pleonastic (see Banes, 2001). Unfortunately, however, while the informal responses of those who tried this experience were apparently very positive (see also Ultrahaptics, 2019), as far as we can tell, no formal testing was conducted. The other thing to note here is how brief the experience was.

Follow-up research by Immotion showed similar results in terms of enhancing user engagement and immersion through the integration of mid-air haptic feedback. The results revealed that haptics increased replays by 20% and all the participants indicated that they would choose a VR experience incorporating these tactile sensations again (Anon., 2019). However, while such results look promising, it may be best to wait on the results of peer-reviewed experimental research before putting too much weight on such industry-sponsored research findings.

## Conclusions

It is intriguing to note how the enthusiastic claims that have appeared in recent years concerning the imminent emergence of haptics in entertainment have yet to materialize.^23^ As this narrative review has attempted to make clear, various technological, financial, creative/artistic, and cognitive/perceptual challenges have hampered the introduction of tactile elements to both public and home entertainment settings (see Spence & Gao, 2024, on the former). However, the growing ubiquity of touch-enabled smartphones, and other wearable devices, offers a possibly unique opportunity to engage the user's sense of touch. This raises the question of whether it is really possible to augment digital storytelling and gaming, especially given the fact that people are consuming more of their digital content on their smartphones than ever before (cf. SkyQuest Technology Consulting Ltd., 2022), and the majority of people use the tactile functionality on a daily basis (Réhman et al., 2008). At the same time, the standardized API protocols that are now being developed mean that it is much easier than ever before to both program and deliver some form of primitive tactile stimulation to users (Blenkinsopp, 2019). The Ultrahaptics company is now producing commercially viable, and seemingly robust, mid-air haptics solutions for the marketplace. That said, the range of vibrotactile stimulation effects is limited to various kinds of primitive vibratory effect delivered from a very limited number of locations (mostly just the palmar surface of the hand holding the device). However, Apple's Taptic API is enhancing the range of tactile stimuli that are possible (see also Sahami, Holleis, Schmidt & Häkkilä, 2008). Given such developments, there would currently appear to be something of a unique opportunity to consider the widespread introduction of a limited range of tactile (what is sometimes referred to as haptic) content to augment the user's experience.

At the same time, however, and as Eid and Osman (2016) note, the price and complexity of haptic interfaces constitute a limitation in terms of their commercial uptake in the context of gaming and entertainment. These researchers also highlight the lack of a range of haptic “keys” to induce emotional changes. It is important to provide robust demonstrations of the value of augmenting experience design with touch (Maggioni et al., 2017). However, it is at this point that one really needs to question what one is trying to achieve by stimulating the skin. According to Israr et al. (2014):

Entertainment content: probably the most direct use of FEs is in augmenting the entertainment experience in games, movies, music, shows, and rides, where realistic representation of events all around the user's body may lead to a deeper sense of immersion and believability. Imagine a swipe or stroke on the back that follows an eerie wail just before a ghost comes into view, or the feeling of debris raining down after an explosion. Perhaps viewers will identify more strongly with a character when they can feel his or her heartbeat quicken at the approach of a loved one, or gradually slow after a chase.

Touch/haptics is always indirect (in the case of its use in cinema), and in need of some degree of interpretation. Furthermore, this review of the literature has highlighted a number of different uses/meanings that researchers have attached to tactile/haptic augmentation (see also Schneider et al., 2017). Moreover, given the many possible uses for, or meanings attached to, various kinds of tactile stimulation, there is likely to be a question as to how the user knows which (kind of) meaning should be associated with any given pattern of haptic stimulation (i.e., is it supposed to be diegetic or not?).

Ultimately, there is likely to be little benefit of pleonastic use of haptics (Banes, 2001), except perhaps a generalized increase in arousal that has often been reported (see Table 2). Note that many tactile solutions require some degree of training in order to be able to interpret the signals (Brewster & Brown, 2004; Réhman et al., 2008). Indeed, although it has often been suggested that adding additional congruent channels of stimulation helps to enhance immersion in, for example, VR applications (e.g., see Hoffman, 1998; Sallnas et al., 2000), it is by no means clear how often this is true, especially in the context of passive (rather than active) tactile/haptic stimulation. Certainly, one might question the claim that “Presence is the magic of VR, and it's almost self-evident that adding the sensation of touch into VR increases users’ sense of presence.” (Blenkinsopp, 2019).

There is also a danger that such tactile stimulation may actually be distracting (Spence & Driver, 1997; Vi et al., 2017), and the nature of tactile experience may be modified by the many multisensory interactions and visual dominance effects that have been documented in the literature (Hutmacher, 2019). It can be argued that too much of the research interest has focused on the question of how to deliver the digital tactile stimulation, and not enough on the question of whether the customer will appreciate the tactile stimulation sufficiently to want to pay for the tactile stimulation device (should that be what is called for), nor on what exactly the tactile/haptic stimulation will be used to convey (see Table 3).

**Table 3.** Different meanings attached to, and effects of, tactile element in film.

Study	Explanation (and problems)	Relevant studies
Pictorial	Conveying visual image on skin (But people struggle to interpret images in part due to insufficient bandwidth)	Bach-y-Rita et al. (1969); Collins (1970); Collins & Saunders (1970)
Metaphorical	Convey metaphorical expressions, such as Butterflies in my stomach’; Interest in semi-intuitive tactile patterns	Brunet et al. (2013); Israr et al. (2014); Lemmens et al. (2009)
Diegetic		
Global	Convey global tactile effects, such as rain or an earthquake/explosion Convey cinematographic effects	Spence and Gao (2024); Clark (2018); Danieau et al. (2014); Réhamn et al. (2008)
Local	Feel object a character touches on screen (Requires consideration of point of view)	O'Modhrain & Oakley (2003); Gallace et al. (2012)
Mood effect	Convey a particular mood (like mood music in film) (The challenge is to determine which tactile patterns are associated with each emotion)	Danieau et al. (2014); Mazzini & Bryan-Kinns (2016); Park et al. (2013)
Arousal	Tactile stimulation can increase arousal	Ablart et al. (2017a, 2017b); Vi et al. (2017)
Presence	Often suggested that adding tactile/haptic feedback increases immersion; But the danger is that it may distract/overload	Hoffman (1998); Sallnas et al. (2000)
Breaking the 4th wall	Occurs with motion platforms; The challenge of point of view, and the distinction between active and passive touch	Spence and Gao (2024); Zhang et al. (2022)

It is interesting to see both the parallel challenges that have limited the introduction of tactile stimulation in the context of public entertainment largely overlapping with those affecting personal tactile augmentation (see Spence & Gao, 2024). At the same time, however, another striking fact is that all of the situations in which the use of tactile augmentation has been tested to date have been in all very short format (i.e., up to a maximum of 10 minutes, but very often shorter than three minutes) (see Table 2). Hence, it will be interesting to see whether fatigue may set in if tactile/haptic augmentation is used over a longer time period (i.e., a whole movie, say, rather than just a scene). It is also worth noting the careful selection of movie clips that might benefit from some form of tactile/haptic augmentation, a key feature of many of the studies reviewed in Table 2.

## Notes

1. ‘Home entertainment’ refers to the use of multiple electronic entertainment devices to create and reproduce a movie theatre experience and mood in the home (Anon, 2023).

2. Haptics is a term that is sometimes used to refer to active touch, while in certain research communities, such as among HCI (human–computer interaction) researchers, it is used as a synonym for touch.

3. Indeed, there is little online concerning Disney's Surround Haptics over the last decade (though see the Marvel Avengers Vybe Haptic Gaming Pad; Workman, 2012). In fact, other companies now appear to have started using the name for a more traditional butt-shaker device, albeit one that is described as a ‘Haptics Ecosystem’ (eeNews Europe, 2019).

4. None of these skin sites, note, are known for their tactile discrimination or information-processing abilities (Gallace & Spence, 2014; Pratt et al., 2024; Weinstein, 1968).

5. One might also consider the butt-shaker type of devices in this category. Though, it is perhaps worth noting that in 2010, Kudo Tsunoda, general manager of Microsoft Game Studios, described the rumble technology incorporated into games controllers since the 1990s as nothing more than ‘a rudimentary form of haptic feedback’ (Tsunoda, 2010, as cited in Parisi, 2018, p. 336).

6. Post-Covid, there may also be concerns about the cleanliness of any public tactile stimulation device that makes direct contact with the skin. This is one of the potential advantages of mid-air haptic stimulation (see also London Metropolitan University, 2018, for other reasons to worry about touching a surface that has also been touched by many other people).

7. As Sally Banes (2001, pp. 68–69) puts it in the context of the use of olfaction in the context of live performance: ‘So often the use of smell seems merely iconic and illustrative, a weak link in a chain of redundancy across sensory channels that does nothing more than repeat what is already available visually and aurally.’ (cf. C. S. Peirce's, 1991, semiotic triad, icon-index-symbol when thinking about how tactile stimulation might be used to either enhance, or else depart from, a realistic rendition).

8. Though, as one commentator noted, when talking about Sony's decision to remove vibration from their PlayStation 3 console, possibly linked to just such patent-related issues (Reimer, 2007): ‘When you think about the investments they’re making in improving graphics and sound, these are all meant to try to immerse you or put you in the middle of gameplay… So to take vibration out of a driving game or a first person shooting game, I can’t imagine how people will be able to view that as an advancement in gaming.’ (quote in Murdey, 2006, p. 4). For more information on the licensing of these technologies, see https://www.philips.com/a-w/about/innovation/ips/ip-licensing/programs/touch-enabled-devices.html.

9. At the same time, the quality of tactile stimulation that is possible nowadays on certain mobile devices is starting to improve noticeably. Consider only the jump in quality between the rumble on the iPhone 6 and the taptic engine on the iPhone 7 (and later generations). Modern VR controllers, like the Privacy Oculus Touch controllers, also include sophisticated Microelectrical Mechanical Systems (MEMS)-based cutaneous feedback systems.

10. Note that such a contrastive function is harder to imagine in the case of tactile/haptic stimulation, given the fact that the meaning of such tactile stimulation typically needs to be inferred from what is going on in the other senses (e.g., vision and audition).

11. This art work from Miho Suginami involved a group of people making plaster casts of their own hands, which the artist then mounted on a wall, whereupon the group involved in the work's creation were invited back to touch the art. Remarkably, albeit only anecdotally, a few people apparently had the disconcerting sensation of recognizing a family member from the touch of their plaster cast hand, thus hinting at the existence of some form of memory that was triggered specifically by touch.

12. These researchers demonstrated that people tended to use weak touches for positive emotions, and hard, fast, and continuous touches for negative emotions.

13. We certainly agree with Israr et al. (2012, p. 1088) when they write that: ‘Designing tactile technologies that enhance the vocabulary of tactile “expressions” is one of the most important research directions in haptics and HCI today’.

14. Scent has sometimes been used to induce a specific mood in the audience in the context of live performance (Banes, 2001). It is, though, much harder to see how the same could be done via tactile stimulation, given that c-tactile afferents, the so-called pleasure system in the human skin, respond preferentially to slow warm stroking (cf. Gallace & Girondini, 2022; Spence, 2022).

15. Though, as noted by Spence and Gao (2024), such effects were sometimes delivered by Sensurround, as in the 1974 movie *Earthquake*. In this case, the ultralow frequency sound waves may have stimulated both the ears and bodies of cinema-goers.

16. Perhaps using the massage function on a business class airplane seat while watching an in-flight movie might count as an incidental non-diegetic haptic effect.

17. ‘Going to the Feelies this evening, Henry?’ enquired the Assistant Predestinator. ‘I hear the new one at the Alhambra is first-rate. There's a love scene on a bearskin rug; they say it's marvellous. Every hair of the bear reproduced. The most amazing tactual effects.’ (Huxley, 1932, p. 39).

18. Note that attempts to represent visual stimuli pictorially by means of spatial patterns of tactile stimulation on the skin surface have not proved particularly successful, despite early optimism amongst some researchers (e.g., see Bach-y-Rita et al., 1969; Collins, 1970; Collins & Saunders, 1970).

19. Note that a similar issue has been identified in the context of scratch-and-sniff books (Spenceet al., 2024).

20. As Hempel and Altinsoy (2005, p. 149) note when considering the potential of haptic interfaces in an entertainment and gaming context: ‘In current driving games and flight simulators, which are the first areas of frequent haptic interaction application, one can achieve very realistic effects. By fighting or shooting, force feedback in different intensities could excite or frighten the user. Haptics could serve as an adrenaline pump (Minsky, 2000).’ At the same time, however, it is also important to note that the context in which the tactile stimulation is delivered (e.g., laboratory vs. in the wild) may also change the user's reaction to tactile augmentation (Desnoyers-Stewart et al., 2024).

21. Qualitative responses included: ‘I loved the sound of that one. It was kind of scary’, another participant focused on the sensation on the hand ‘It was strange, it freaked me out because I wanted to pull my hand out [from the plinth] but I didn’t want to because I wanted to carry on and see what it was like.’ And ‘I’m speechless about that one. It made me goose bumpy’; ‘I loved it, I wanted to keep my hand in there. I loved feeling what the painting looks like and feeling the empty space and the negative space and then trying to relay that feeling onto the painting when I was looking at it.’ (both quotes from Vi et al., 2017).

22. However, although such mixed reality interventions are promising (Desnoyers-Stewart et al., 2024; Pells, 2015), they appear impractical for/in the home environment.

23. For example, Kyle Melnick for VR Scout suggested that ‘2018 is shaping up to be the year of haptic feedback’.
